# Involvement of transmembrane protein 184a during angiogenesis in zebrafish embryos

**DOI:** 10.3389/fphys.2022.845407

**Published:** 2022-09-02

**Authors:** Cassandra J. Field, Alexis M. Perez, Taryn Samet, Victoria Ricles, M. Kathryn Iovine, Linda J. Lowe-Krentz

**Affiliations:** Department of Biological Sciences, Lehigh University, Bethlehem, PA, United States

**Keywords:** heparan-binding protein, heparan sulfate proteoglycans, vascular endothelial growth factor, TMEM184A, angiogenesis

## Abstract

Angiogenesis, the outgrowth of new blood vessels from existing vasculature, is critical during development, tissue formation, and wound healing. In response to vascular endothelial growth factors (VEGFs), endothelial cells are activated to proliferate and move towards the signal, extending the vessel. These events are directed by VEGF-VEGF receptor (Vegfr2) signal transduction, which in turn is modulated by heparan sulfate proteoglycans (HSPGs). HSPGs are glycoproteins covalently attached to HS glycosaminoglycan chains. Transmembrane protein 184a (Tmem184a) has been recently identified as a heparin receptor, which is believed to bind heparan sulfate chains *in vivo*. Therefore, Tmem184a has the potential to fine-tune interactions between VEGF and HS, modulating Vegfr2-dependent angiogenesis. The function of Tmem184a has been investigated in the regenerating zebrafish caudal fin, but its role has yet to be evaluated during developmental angiogenesis. Here we provide insights into how Tmem184a contributes to the proper formation of the vasculature in zebrafish embryos. First, we find that knockdown of Tmem184a causes a reduction in the number of intact intersegmental vessels (ISVs) in the zebrafish embryo. This phenotype mimics that of *vegfr2b* knockout mutants, which have previously been shown to exhibit severe defects in ISV development. We then test the importance of HS interactions by removing the binding domain within the Tmem184a protein, which has a negative effect on angiogenesis. Tmem184a is found to act synergistically with Vegfr2b, indicating that the two gene products function in a common pathway to modulate angiogenesis. Moreover, we find that knockdown of Tmem184a leads to an increase in endothelial cell proliferation but a decrease in the amount of VE-cadherin present. Together, these findings suggest that Tmem184a is necessary for ISVs to organize into mature, complete vessels.

## Introduction

Studies of developmental angiogenesis in mice and zebrafish, among other model organisms and tissue models, have resulted in a good understanding of the developmental timing, vessel patterning, cellular compositions, and control mechanisms of angiogenesis (reviewed in Geudens and Gerhardt, 2011; Herbert and Stainier, 2011). While many specific signaling systems that control the process have been characterized (Herbert and Stainier, 2011), new details of the signaling systems continue to be identified (e.g., Corti et al., 2019). The zebrafish, *Danio rerio*, is one commonly used model to study vascular development as the embryos are completely transparent during embryogenesis, and development to adulthood occurs in about 2–3 months (e.g., Higashijima, 2008). Moreover, a reporter line has been established (Lawson and Weinstein, 2002). Transgenic zebrafish, expressing EGFP driven by the fli1 endothelial cell (EC) marker, express EGFP specifically in ECs. This aids in the visualization of cell migration and change during vascular development (Lawson and Weinstein, 2002).

Vasculogenesis begins in zebrafish at 14 h post fertilization (hpf) when the first population of angioblasts within the lateral plate mesoderm begins to migrate towards the embryonic trunk midline (Gavard and Gutkind, 2006; Geudens and Gerhardt, 2011; Helker et al., 2013). This is followed by angiogenesis, the process by which new vasculature develops from existing vessels (Geudens and Gerhardt, 2011; Gore et al., 2012; Chiodelli et al., 2015). Angiogenesis is promoted through EC proliferation, EC migration toward angiogenic signals, adjustments in permeability to allow for tissue growth, and EC survival (reviewed in Herbert and Stainier, 2011; Koch et al., 2011; Chiodelli et al., 2015; Simons et al., 2016).

During developmental angiogenesis in zebrafish, sprouts begin to deploy from the dorsal aorta in a carefully choreographed manner, forming a regular array of ISVs (Childs et al., 2002). The aortic migrant cells will then follow the same track, forming the stalk of the vessel (Gavard and Gutkind, 2006; Hasan et al., 2017). These stalk cells elongate and are held together by vascular endothelial cadherin/Cadherin 5 (VE-cadherin/Cdh5) junctions, responsible for cell-cell adhesion. By 30 hpf, there is a pattern of VE-cadherin extending along the vessel’s axis (Blum et al., 2008). As ISV development continues, the tip cell will eventually reach the dorsal side of the embryo (Blum et al., 2008). The stalk cells follow behind, ultimately contributing to the formation of the dorsal longitudinal anastomotic vessel (DLAV) (Blum et al., 2008). As these stalk ISVs extend, the VE-cadherin facilitates the elongation (Sauteur et al., 2014) and is involved in initiating interactions between cells from adjacent ISVs leading to completion of the ISVs and anastomosis and resulting DLAV production (Sauteur et al., 2017). One convenient aspect of using ISV development in zebrafish to study signaling within and between endothelial cells is that the mural cell recruitment to the trunk vessels is relatively slow, resulting in few VSMC or pericytes associated with the DA before 72 hpf (Santoro et al., 2009; Whitesell et al., 2014). Using live imaging, the appearance of some mural cells on the ventral portion of the DA began about 36 hpf (Ando et al., 2016), but a requirement for Pdgf signaling for ISV development that did not depend on mural cells has been noted (Wiens et al., 2010). ISV sprouting starts around 24 hpf, and there is no evidence for pericytes or VSMCs on the ISVs until after the vessels anastomose forming the DLAV (Whitesell et al., 2014).

Angiogenesis is significantly driven by VEGFs. In the zebrafish, VEGFs are initially expressed in the medial region of the somites along with several brain regions (Liang et al., 1998). A crucial VEGF during angiogenesis is VEGF-A (Koch et al., 2011). When a VEGF-A ligand binds to Vegfr2, the complex of which is stabilized by HS, the receptor dimerizes resulting in a conformational change that activates it (Koch et al., 2011). At this point, Vegfr2 phosphorylates specific intracellular tyrosine residues, transducing signals in the cell cytoplasm that result in an increase in the EC proliferation, migration, and survival, which are all required during angiogenesis (Koch et al., 2011). Ligand binding by Vegfr2 also triggers endothelial Notch/Delta signaling, which in turn signals cell differentiation and outgrowth from the dorsal aorta (Hasan et al., 2017) through limiting tip cell development and branching (Leslie et al., 2007; Siekmann and Lawson, 2007; Suchting et al., 2007).

Interactions of HSPGs with growth factors (including VEGF) and their receptors have been shown to be crucial in vascular development reviewed in (Herbert and Stainier, 2011; Chiodelli et al., 2015). HSPGs, consisting of a core protein attached to heparan sulfate (HS) side chains, are ubiquitously found at the cell surface in nearly all eukaryotic cells (Bernfield et al., 1999). Despite the large number of HS binding proteins, the detailed mechanisms by which heparin and HS initially alter angiogenesis are not completely understood. Prior studies (Chen et al., 2004) identified the HSPG Syndecan-2 (Sdc2) as required for vascular development in zebrafish. Evidence for roles of Sdc1 in mice (Beauvais et al., 2009) and Sdc4 (Echtermeyer et al., 2001; Nunes et al., 2008; Baeyens et al., 2014; Johns et al., 2016) have also been found. Recent work has identified VEGFA/VEGFR2 specific requirements for Sdc2 that depend upon Sdc2 protein-induced specific sulfation changes (Corti et al., 2019).

While HS effects have often been presumed to depend solely on HS interactions with growth factors and their receptors, numerous studies have suggested that a receptor for heparin plays a role in these responses as well. For example, treatment with exogenous heparin has been used to identify novel heparin/HS binding proteins (Sarrazin et al., 2011; Schultz et al., 2017). The heparin receptor, Tmem184a, was identified in vascular cells (Farwell et al., 2016; Pugh et al., 2016) with a putative heparin binding domain at the C-terminal region of the protein. Heparin binds specifically to Tmem184a which is required for anti-proliferative signaling cascades induced by heparin treatment of vascular cells *in vitro* (Gilotti et al., 2014; Farwell et al., 2016; Pugh et al., 2016; Li et al., 2020).

Tmem184a has been characterized *in vivo* in the adult regenerating zebrafish fin. Interestingly, Tmem184a-knockdown (KD) in the regenerating fin caused reduced vascular outgrowth and reduced regenerate length, but an increased number of ECs and disorganized vessels (Farwell et al., 2017). These findings suggest that Tmem184a function is required to limit the proliferation of ECs and thereby modulate vessel outgrowth during regeneration. Here, we provide evidence that Tmem184a functions similarly during development. We find that morpholino (MO)-mediated KD of Tmem184a significantly decreases the number of intact ISVs in an embryo, mimicking *vegfr2b* phenotypes (Habeck et al., 2002; Bahary et al., 2007; Covassin et al., 2009; Villefranc et al., 2013) while decreasing embryo survival and demonstrate that reduced Tmem184a and Vegfr2b synergize during ISV outgrowth.

Tmem184a binds exogenous heparin *in vitro* and is responsible for anti-proliferative signaling cascades induced by heparin (Farwell et al., 2016; Pugh et al., 2016; Farwell et al., 2017). We hypothesize that it normally interacts with HS chains in cells. Here we report that Tmem184a has similar effects in developing zebrafish embryos. Tmem184a KD-reduced ISV outgrowth is accompanied by increased EC proliferation, and a decrease in the amount of VE-cadherin expression responsible for EC adhesion. The putative heparin binding domain is crucial for Tmem184a effects on angiogenesis. The carboxyl terminal region containing the putative heparin binding domain is crucial for the function, and increased expression of Tmem184a lacking this region results in a dominant negative effect on ISV outgrowth. These findings are consistent with our previous *in vitro* studies and Tmem184a function during fin regeneration angiogenesis.

## Materials and methods

### Statement on the ethical treatment of animals and zebrafish care, housing and husbandry

This study was performed in accordance with the recommendations in the Guide for the Care and Use of Laboratory Animals of the National Institutes of Health. This protocol was approved by Lehigh’s Institutional Animal Care and Use Committee (IACUC) (Protocol # 222, initially approved 4/13/2017 and more recently as protocol 172 approved 10/8/21). Lehigh University’s Animal Welfare Assurance Number is A-3877-01. All experiments were conducted to minimize pain and discomfort. All zebrafish were housed in a re-circulating system built by Pentair, in 3L tanks with up to 12 fish per tank. The fish room was on a 14:10 light: dark cycle with a room temperature maintained at 27–29°C. Water quality was monitored automatically and dosed to maintain conductivity (400–600 μs) and pH (6.95–7.30). Nitrogen levels were maintained by a biofilter and a 10% water change was performed daily. Re-circulating water was filtered sequentially through pad filters, bag filters, and a carbon canister before circulating over ultraviolet lights for sterilization. Fish were fed three times a day, once with brine shrimp (hatched from INVE artemia cysts) and twice with flake food (Zebrafish Select Diet, Aquaneering Inc.).

### Zebrafish strains

The zebrafish C32 strain described previously (Rawls et al., 2003) was used as the animal model for this study. The *Tg* (*fli1:egfp*) transgenic line (as described in Lawson and Weinstein, 2002) was employed for this study. Both males and females were utilized.

### Morpholino injection

MOs purchased from GeneTools, LLC (Philomath, OR) were dissolved in sterile ddH₂O, at a 1 mM concentration. MOs were heated to 65°C prior to use. All MOs were tagged with either fluorescein or lissamine rhodamine. Two non-overlapping MOs against Tmem184a, published previously (Farwell et al., 2017), were used: one that was an ATG translational start site blocker (5′-CTG​AGA​GTA​GTT​TCA​TTC​ATC​CTG​A-3′) and one that was a splice site blocker between exon two and three (5′-AAA​CAG​GCA​CAC​TCA​CTG​AAT​GGG​C-3′). One MO was used against Vegfr2 (5′-CCG​AAT​GAT​ACT​CCG​TAT​GTC​ACT​T-3′). This MO has been previously published and confirmed for Vegfr2 in (Habeck et al., 2002). A fluorescein labeled “standard control” (SC) MO was used as a control (5′-CCT​CTT​ACC​TCA​GTT​ACA​ATT​TAT​A-3′). Microinjections were performed at the 1 cell stage using the Narishige IM 300 Microinjector and Nikon SMZ 800 for visualization. Embryos were injected with a standard dose of 1 mM unless noted otherwise. Zygotes were sorted for viability and fertilized embryos were kept in an egg water and Ampicillin solution overnight at 28°C. Embryos were then sorted for vascular expression of GFP using the Nikon Eclipse 80i fluorescence microscope. GFP-positive (GFP +) embryos were grown to 48 h, de-chorionated using pronase, fixed in 4% paraformaldehyde (PFA), and kept at 4°C overnight. Embryos were then stored in phosphate buffered saline (1x PBS) at 4°C in the dark for long term use. Survival of all embryos was recorded, and survival plots are based on survival of treated embryos vs. survival of SC injected embryos. SC injected embryos typically survive at 89% of uninjected siblings, likely due to some damage during injections.

### Embryo lysates and immunoblotting

MO-injected embryo lysates were made at 48 hpf for Tmem184a knockdown and standard control. Embryos were de-chorionated with pronase and transferred to a dish containing ice cold de-yolking solution composed of phenylmethylsulfonyl fluoride (0.3 mM PMSF), Ethylenediaminetetraacetic acid (0.5 mM EDTA), and Ringer’s solution. Yolks were removed by pipetting up and down with a P200 pipette and monitored using the Nikon SMZ 800 microscope. The de-yolked embryos were washed twice with ice cold Ringer’s solution and excess liquid was removed. The embryos were suspended in sterile water (1 µL/embryo) and flash frozen in liquid nitrogen. They were kept at -80°C until the next step. After thawing on ice, they were homogenized in 2X-SDS sample buffer at the ratio of 2 µL/embryo, and then sonicated for 10 s and boiled for 5 min. Lysate samples were stored at -80°C and boiled again before use. Alternatively, single embryos were prepared using essentially the method of (Schnabel et al., 2019). Three embryos per treatment were combined in 20 µL of embryo buffer with protease inhibitor cocktail, homogenized and mixed with 5X sample buffer before boiling and running on a gel. Protein expression was evaluated using fluorescent western blotting as described previously in (Farwell et al., 2016). A primary antibody specifically for zebrafish was used to detect VE-cadherin (1:2000, GenScript). This polyclonal antibody was generated against amino acids 186-372. Alexa 647 anti-rabbit (1:5000, Jackson Immuno Labs #711 605 152, RRID:AB_2492288) was used to detect VE-cadherin primary antibody. Mouse anti-tubulin (1:10000, Abcam #7291, RRID:AB_2241126) was used to confirm equal sample loading. Alexa 488 anti-mouse (1:5000, Jackson Immuno Labs #715-545-150, RRID:AB_2340846) was used to detect the tubulin primary antibody. Secondary antibodies are affinity purified to reduce cross-species interactions. ImageJ was then used to measure band intensities as described in (Banerji et al., 2016).

### Imaging analysis of embryos

Fixed embryos were mounted on double cavity slides using either 3% methyl cellulose or 1% low melt agarose for embedding. After the embryos were oriented from head (left) to tail (right), they were covered with a coverslip. The vascular phenotype was observed using either the Nikon C2+ confocal microscope or Zeiss LSM 880 confocal microscope. Z-stack and still images were taken for analysis. Z-stacks of the whole embryo were taken to ensure to that vascular growth was mirrored for the dorsal aorta and cardinal vein. These images were then analyzed to quantify the number of intact intersegmental vessels along the trunk and tail of the embryo. For consistency, completed ISVs were counted starting three from the head down to the cloaca. Most images are shown as maximum intensity projections of one half of the fish (thereby including one of the two paired ISVs), or as maximum intensity projections of the entire fish if so noted. Only the ISVs that had completed growth up to the DLAV were considered intact. *ImageJ* was used to measure the distance between ISV.

### mRNA treatments

Full length mRNA (i.e., 5′UTR plus coding sequence) encoding 420 amino acids and *tmem184a-ΔCT* mRNA (i.e., full length minus coding for the final 100 amino acids—referred to as the C-terminal domain) for Tmem184a were synthesized in the pcDNA3.+ plasmid by GenScript. For each sequence, the plasmid DNA was then linearized by performing an AvrII digest (NEB). The Invitrogen mMessage mMachine kit was used to generate capped *tmem184a* mRNA and *tmem184a-ΔCT* mRNA. The concentration was assessed using the Thermo Scientific Nanodrop 2000; size and integrity of the mRNA was evaluated on a formaldehyde gel and imaged. The mRNAs were diluted to a concentration of 25 ng/μL or 100 ng/μL with phenol red for visualization. This was heated at 65°C for 5 min and then injected into zebrafish embryos at the 1 cell stage as described above. The 25 ng/μL mRNA or 100 ng/μL were also co-injected into embryos that had been injected with the Tmem184a splice blocking morpholino (i.e., which does not target the synthesized mRNA). Both the mRNA injected embryos and co-injected rescue embryos were fixed in 4% PFA overnight at 48 hpf and then ISV growth was analyzed using the Nikon C2+ confocal microscope, ×4 objective to observe the entire embryo and ×16 objective to observe and analyze ISVs growth within the trunk (defined as starting three ISVs from head to the cloaca).

### PH3 cell proliferation assay

Cell proliferation in 48 hpf GFP + zebrafish embryos was measured by immunostaining for histone-3-phosphate (PH3) as described previously for fins (Farwell et al., 2017). Embryos were washed in 1x PBS (3 × 5 min) in a 24 well plate, followed by blocking with PH3 buffer (2% BSA/1x PBS, BSA, 0.01% TX100) for 1 hr. The embryos were placed in primary antibody (anti-PH3 rabbit (1:100, Millipore 06-570, RRID:AB_310177) and mouse anti-GFP (1:200, Santa Cruz sc9996, RRID:AB_627695) made in 2% BSA/2xPBS and incubated in a dark room overnight (4°C). The anti-GFP antibody ensured that sufficient staining was present to discriminate nuclei in the ECs from other nuclei in the embryo. The following day, the embryos were washed with PH3 blocking buffer 3 × 5 mins. The embryos were then placed in secondary antibody (Alexa 647 anti-rabbit (1:200, Jackson Immuno Labs #711 605 152, RRID: AB_2492288) and TRITC anti-mouse (1:200, Jackson Immuno Labs #715-025-151, RRID: AB_2340767) made in 2% BSA/2x PBS and incubated in a dark room overnight (4°C). Secondary antibodies were purchased as affinity purified to reduce cross-species interactions. Embryos were washed 3 × 5 min in 1x PBS. PH3 stained Tmem184a KD embryos, and PH3 stained standard control embryos were imaged for EGFP and PH3 with the ×10 objective of the Nikon C2+ confocal microscope. Z-stacks were taken of each embryo image. To analyze the PH3-positive cells in Fli1-positive endothelial cells, PH3 was pseudocolored to red and EGFP was pseudocolored to green for all embryos. The Z-stacks were projected in 3D and individual PH3/Fli1-positive nuclei were confirmed by rotating in all directions. The positive cells showed co-localization of PH3 and Fli1 in yellow. These data were quantified by observing the images and calculating the percent of ISVs containing an PH3-stained nucleus (Farwell et al., 2017).

### Immunofluorescence of VE-cadherin cdh5 in zebrafish embryos

To stain VE-cadherin in the zebrafish, a polyclonal antibody was generated in rabbit (GenScript) against zebrafish VE-cadherin (amino acids 186-372). This peptide was used to confirm specific peptide binding of the antibody in Westerns. Tmem184a ATG MO and SC MO zebrafish embryos fixed at 43 hpf were utilized. Two to four embryos were stained per trial. Each embryo was placed in a different well, in a twenty-four well plate. They were first washed in 1xPBST (1xPBS/0.1% Tween20) 4 × 5 min, removing the embryo each time after a wash and placing it in a new well using a glass pipet. A fifth wash was done for 1 hour in 1xPBSTX (1xPBS/0.1%Tween/0.1% Triton). The embryos were then blocked for 1 h (1xPBSTX/10% BSA). Subsequently, the zebrafish embryos were placed in primary antibody solution (VE-cadherin 1:200, GenScript) diluted in 1xPBSTX 1% BSA and incubated in a dark room at 4°C overnight. The following day the zebrafish embryos were washed 5 × 10 min and 3 × 1 h with 1xPBSTX 1% BSA. Afterward, the embryos were placed in a secondary antibody solution (Alexa-647 anti-rabbit 1:1,000, Jackson Immuno Labs #711-605-152, RRID: AB_2492288), made in 1xPBSTX 1% BSA and incubated in a dark room at 4°C overnight. The following day, the embryos were washed for 4 × 5 min in 1xPBST and then mounted onto double cavity slides for imaging on the Zeiss LSM 880 at ×20 magnification. Z-stacks were taken for both arterial and venous intersegmental vessels separately. Maximum intensity projections were then utilized to better visualize the ISVs, and intensity for each ISV was determined. Intensities of individual cell to cell adhesions within the ISVs were also determined.

### Statistical analysis

All data shown are presented as the mean ± standard error of the mean. A two-tailed unpaired t-test was used to determine if there was a statistically significant difference in experiments that compared two groups, and an ANOVA test followed by a Tukey post hoc test was used to compare multiple groups. A power test analysis was used to determine the necessary sample size (n) to ensure we had true significance. Only values giving *p* < 0.05 are reported as significant. The statistical software used for analyzing the data was *Prism8.*

## Results

### Evidence for Tmem184a roles during developmental angiogenesis

**FIGURE 1:**
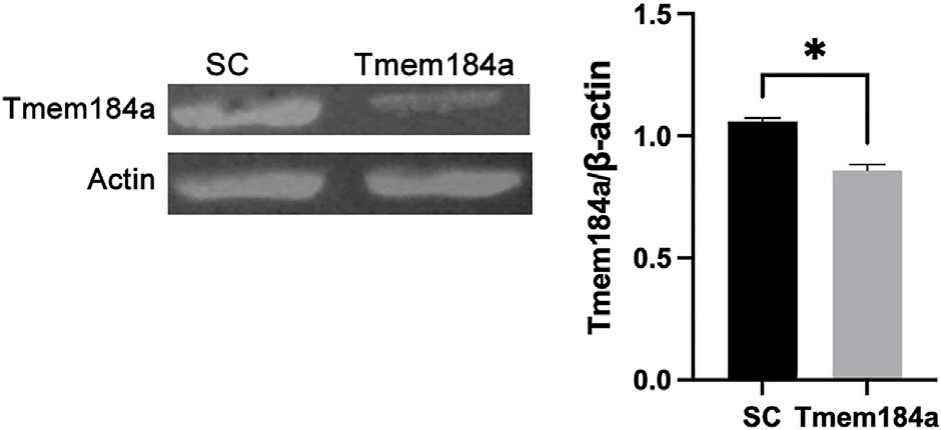
Tmem184a MO Treatment Results in Tmem184a Protein Decrease. Embryos were injected with SC or Tmem184a ATG MO and harvested for western blotting. Three identically treated embryos constituted a single sample. One of three identical experiments is shown with the graph summarizing the data.

Our prior studies on the role of Tmem184a during angiogenesis in regenerating fins provided evidence that *in vivo*, Tmem184a is expressed only in endothelial cells, whether before amputation or during regeneration (Farwell et al., 2017). To confirm that the MO treatment of embryos also results in knockdown of Tmem184a in development, we identify consistent decreases in Tmem184a protein in embryos treated with Tmem184a MO (Figure 1).

### Tmem184a KD results in decreased survival and a decreased number of completed intersegmental vessels

**FIGURE 2:**
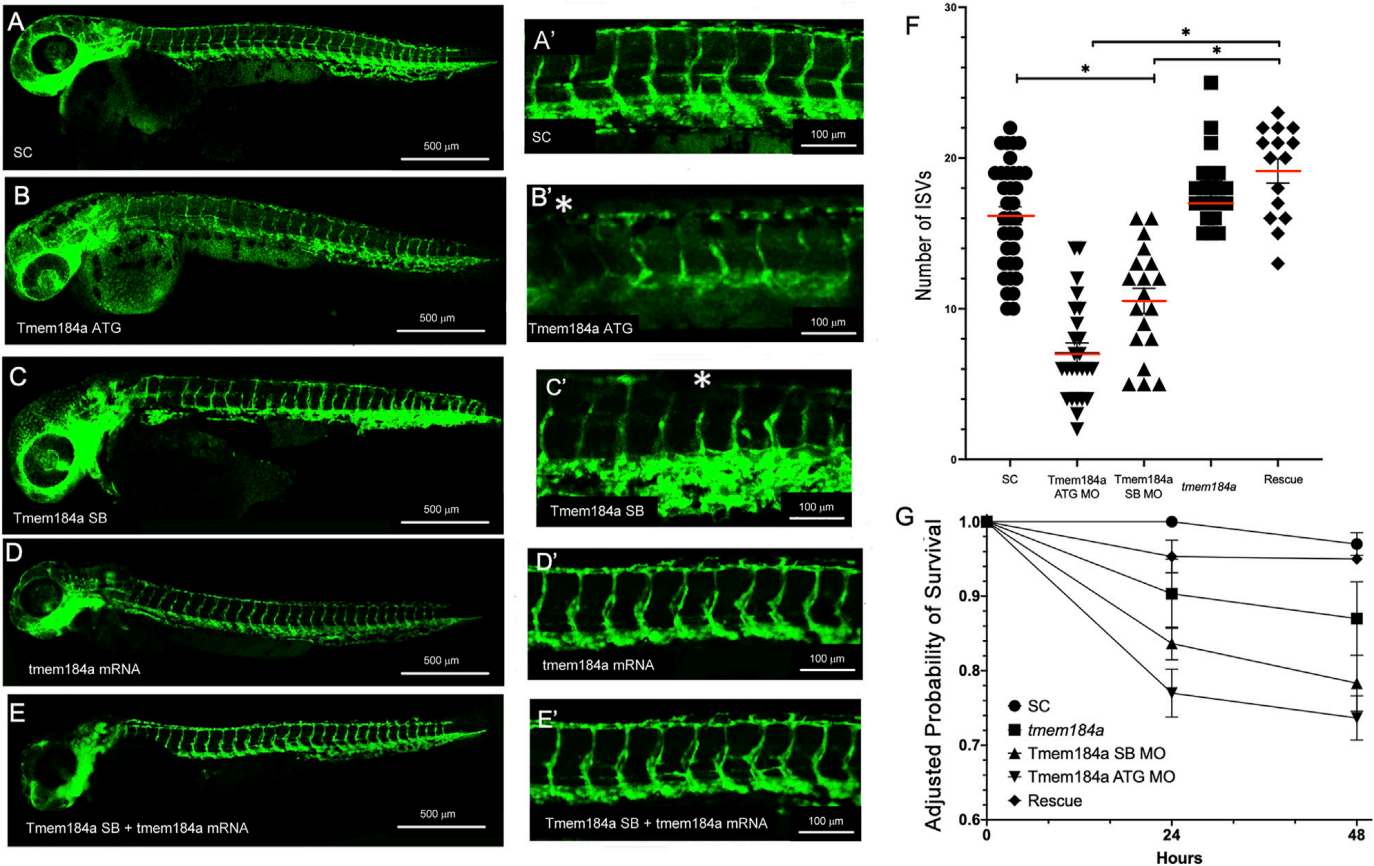
Tmem184a KD Results in Decreased Number of Intact Intersegmental Vessels. **(A,A′)** SC MO injected embryo (n = 35). **(B,B′)** Tmem184a SB MO injected embryo (n = 25). **(C,C′)** Tmem184a ATG MO injected embryo (n = 19). Asterisks indicate ISVs that failed to complete injected embryos. **(D,D′)** Injection with full-length Tmem184a mRNA alone (n = 21). **(E,E′)** Co-injection with full-length Tmem184a mRNA rescues vascular defects in Tmem184a SB MO injected embryos (n = 15). **(A′–E′)** illustrate zoomed trunk regions of the fish. **(F)**. KD by SB and ATG MOs compared to SC MO embryos and rescue embryos with SB MO and Tmem184a mRNA, illustrating the mean number of intact ISVs ± SEM. *p* < 0.001. **(G)** Embryo survival for all treatments shown as compared to SC embryo survival at 24 h ± SEM *p* < 0.001.

In order to better understand the role of Tmem184a during angiogenesis in the zebrafish embryo, we performed MO-mediated KD of the protein and then observed the resulting vascular phenotypes in embryos with decreased levels of Tmem184a. KD was performed using two non-over-lapping MOs, including an ATG blocker, which blocks the translational start site, and a splice blocker (SB), which inhibits pre-mRNA processing. We also used a standard control (SC) MO for comparison. The ATG MO, SB MO, and SC MO were injected into embryos at the 1 cell stage using the *Tg* (*fli:egfp*) zebrafish transgenic line. To determine whether Tmem184a KD had any detrimental effect on trunk vasculature, we imaged MO-injected *Tg* (*fli:egfp*) -positive embryos at 48 hpf (Figures 2A–E). Similar to uninjected siblings, embryos injected with the SC MO did not exhibit any defects in vascular development. Tmem184a MO KD using either targeting MO resulted in significantly fewer intact ISVs as compared to embryos injected with the SC MO (Figures 2A,A’ vs 2B,C). Only vessels that successfully completed outgrowth to converge with the DLAV were considered intact. Without normal levels of Tmem184a expression, ISVs began to sprout similarly to control embryos, but often did not complete outgrowth by 48 h, resulting in shortened vessel length and an incomplete DLAV. On average, ATG MO injected embryos had only 7 intact ISVs in the trunk and SB MO injected embryos had 11, whereas SC embryos have 16 (Figure 2F), suggesting that Tmem184a is required for proper angiogenesis.

We previously demonstrated the specificity of both Tmem184a MOs targeting the Tmem184a protein in the regenerating zebrafish fin model (Farwell et al., 2017). To further confirm this specificity, we tested mRNA rescue of the Tmem184a KD to demonstrate that the observed phenotypes are due to reduced Tmem184a protein. We prepared full length, capped mRNA. Embryos were injected with the Tmem184a SB MO (i.e., which does not target the *in vitro* synthesized *tmem184a* mRNA), followed by an injection of 25 ng of mRNA. We found that there was a significantly greater number of intact ISVs in the doubly injected embryos as compared to embryos injected with the Tmem184a SB MO alone (Figures 2E vs 2C). Therefore, the *tmem184a* mRNA rescues the defective vessel phenotype and restores vessels to an intact length. The *tmem184a* mRNA alone had no significant effect on the ISV phenotype (such as induction of additional vasculature) when injected into embryos not also subjected to the Tmem184a MO (Figure 2D).

We also note that Tmem184a targeting MOs impact survival (Figure 2G). At 24 h, the KD embryos survived slightly less often than SC MO treated embryos, and survival of the KD embryos decreased further at 48 h. These results suggest that the Tmem184a KD treatment had a negative impact on embryo survival beyond the angiogenesis effects we monitored. The number of survivors in KD embryos did not change dramatically after 48 h, at which point, MO efficacy may be reduced (i.e., regenerating caudal fin vasculature eventually recovered and grew out over time). Importantly, survival of KD embryos that also received the *tmem184a* mRNA matched the SC MO embryo survival (Figure 2G) further supporting the idea that the Tmem184a KD was detrimental to the embryo survival (i.e., since replacing the Tmem184a by providing mRNA to direct synthesis of the intact protein countered the KD effects). We note that the survival of the *tmem184a* mRNA injected embryos that were not also injected with any MO was slightly lower than the control or rescue embryos in this assay, but was significantly higher than either KD treatment. Therefore, there might also be an impact of too much Tmem184a protein in early embryos, but we did not observe evidence of that in the trunk vasculature.

We further demonstrated MO specificity by testing the Tmem184a ATG and SB MOs for synergistic effects when co-injected at sub-threshold dosages. Thus, we employed MO concentrations that showed limited defects in vascular outgrowth and increased survival for both the Tmem184a ATG and SB MOs (Supplementary Figures S1A–D). If both the Tmem184a ATG and SB MOs share a common target (i.e., *tmem184a*), more severe phenotypes are expected in embryos injected with both 0.50 mM ATG MO and 0.75 mM SB MO. Indeed, we found that the number of intact ISVs in the co-injected embryos was smaller than expected compared to the simple additive effect of the two MOs (Supplementary Figure S1E). Further, the two subthreshold MOs together resulted in lower survival than the additive effect of the two subthreshold MOs (Supplementary Figure S1F). These findings provide evidence for synergy, signifying that both non-overlapping ATG and SB MOs directly target *tmem184a*, rather than producing a phenotype caused by off-target effects. The evidence that these MOs decrease Tmem184a protein levels (Figure 1 and our earlier work), in addition to the successful *tmem184a* mRNA rescue of MO-induced phenotypes and demonstrated evidence for synergy of the ATG and SB MOs provide strong support for the conclusion that Tmem184a is required during angiogenesis.

### Removing Tmem184a HS binding domains negatively affects angiogenesis

**FIGURE 3:**
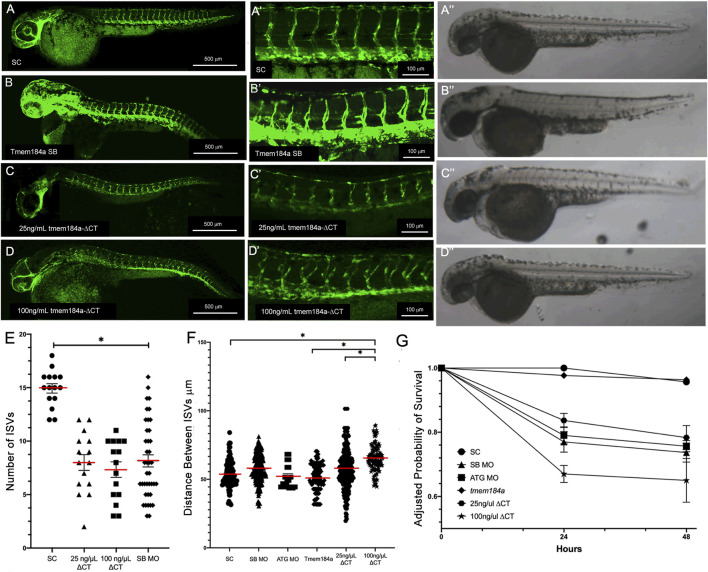
Removing HS binding domains negatively affects Angiogenesis. Embryos were injected with **(A,A′)** SC MO (n = 20) **(C,C′)** 25 ng/μL *tmem184a ΔCT* (n = 60) or **(D,D′)** 100 ng/µL *tmem184a ΔCT* (n = 60) and evaluated at 48 hpf as described in Methods. SB results are shown for comparison **(B,B′)**. **(A′′–D′′)** illustrate brightfield images from identically treated embryos. Graph **(E)** illustrates the intact ISVs in SC, *tmem184a -ΔCT* at either concentration. *p* < 0.001 **(F)** The distance between ISVs was measured for each treatment using Image J. P < 0.001 **(G)** Embryo survival for all treatments illustrated relative to 24 h survival of SC embryos.

Because Tmem184a was identified as a heparin receptor, it was important to determine whether the role of Tmem184a in vascular effects depended on its ability to interact with heparin and/or HS chains. We previously identified a putative heparin/HS binding location near the carboxyl terminus of Tmem184a (Pugh et al., 2016). To test the importance of this putative HS binding domain in Tmem184a in developmental angiogenesis, we designed and studied an mRNA variant of *tmem184a* lacking the sequence coding for its C-terminal domain and HS binding site (i.e., *tmem184a-ΔCT*) (see Figures 3A–F). Unlike the full length *tmem184a* mRNA, the *tmem184a-ΔCT* alone had significant negative impacts on survival similar to the MOs (Figure 3G). Embryos injected with 25 ng/μl *tmem184a-ΔCT* showed abnormal ISV development (Figure 3E). The average number of complete ISVs present at 48 h in the 25 ng/μL *tmem184a-ΔCT* injected embryos was significantly lower than in the embryos that were injected with the standard control MO, and significantly different from injection of 25 ng/μl of the full length *tmem184a* mRNA (Figures 3C,C′). Brightfield images with these treatments do not illustrate differences indicating additional changes in the fish. Increasing the concentration of injected *tmem184a-ΔCT* mRNA to 100 ng/μL further decreased survival, but had a similar effect on the number of completed ISVs (Figures 3D,D’). In addition, we noted an increased lateral distance between ISVs following injection of both doses of *tmem184a-ΔCT* mRNA (Figure 3F). The 25 ng/μl dose increased the distance between ISVs to about 58 μm (i.e., compared to about 51–53 μm for either the SC MO or the full length *tmem184a* mRNA at 25 ng/μL). The 100 ng/μL dose further increased the distance between ISVs (Figure 3F). These results indicate that the expression of the Tmem184a-ΔCT variant inhibits the function of endogenous Tmem184a, and further suggests that the HS binding domain is important for fish survival and in modulation of ISV development.

**FIGURE 4:**
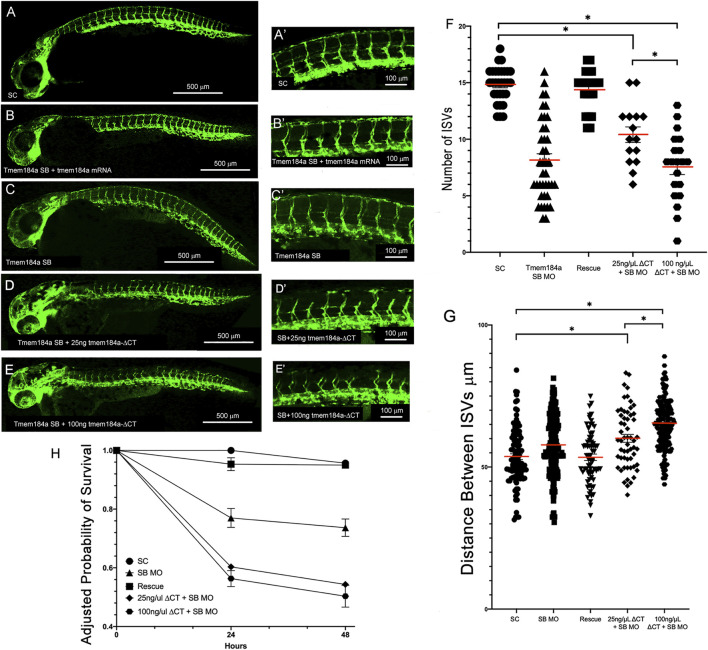
*tmem184a-∆CT* mRNA does not rescue knockdown phenotypes. Embryos were injected with **(A,A′)** SC MO (n = 20) **(B,B′)** SB MO followed by 25 ng/μL full-length Tmem184a mRNA (n = 60) **(C,C′)** SB MO (n = 40) **(D,D′)** SB MO followed by 25 ng/μL *tmem184a-ΔCT* (n = 60) or **(E,E′)** SB MO followed by 100 ng/µL *tmem184a-ΔCT* (n = 60) and evaluated at 48 hpf as described in Methods. **(F)** The graph illustrates the intact ISVs in SC, rescue (SB MO plus full-length Tmem184a mRNA as in Figure 2) embryos, SB MO plus *tmem184a-ΔCT* at either concentration. *p* < 0.001. **(G)** The distance between ISVs was measured for each treatment using Image J. P < 0.001. **(H)** Embryo survival for all treatments illustrated relative to 24 h survival of SC embryos.

We then tested whether the *tmem184a-ΔCT* mRNA could contribute to the rescue of the KD phenotypes of the SB MO despite causing negative impacts when injected alone (Figures 4A–E). Embryos were first injected with the Tmem184a SB MO followed by an injection of *tmem184a-ΔCT* mRNA. In embryos that were doubled injected with 25 ng/μL of *tmem184a-ΔCT* and SB MO, the average number of intact ISV was about 10, similar to the SB MO alone and the 25 ng/μL alone (Figure 4F). Moreover, embryos that were double injected with 100 ng/μL *tmem184a-ΔCT* and SB MO the average number of intact ISVs decreased further, suggesting that increasing the dose of the *tmem184a-ΔCT* mRNA had a negative impact on angiogenesis when combined with Tmem184a KD. These results also indicated that the truncated Tmem184a could not rescue the Tmem184a KD. These results are in striking contrast with the injections of the full length *tmem184a* mRNA in SB MO treated embryos, which instead rescues the number of ISVs to wild-type levels (i.e., as shown for comparison in Figures 4F,G). In addition, injection of the *tmem184a-ΔCT* mRNA with the SB MO decreases survival to around 50% of the SC survival (Figure 4H). These data suggest that the heparin/HS binding domain is critical for the function of Tmem184a in angiogenesis, and also decreases embryo survival, possibly through the effects on angiogenesis.

In embryos that were injected with *tmem184a-ΔCT* after the SB Tmem184a MO, we also saw the distance between ISVs increase compared to embryos that were injected with *tmem184a-ΔCT* alone. To ensure the change is not an artifact of the SB MO we measured fish with the SB Tmem184a MO injections alone and found the average distance between each ISV was unchanged compared to the SC and ATG MOs (Figure 4G). The increased length between ISVs was not part of an overall significant increase in length of these fish. These results using *tmem184a-ΔCT* provide insight regarding the Tmem184a modulation of angiogenesis by indicating the critical nature of the probable HS binding domain*.*

### Tmem184a and Vegfr2 function synergistically to modulate angiogenesis

**FIGURE 5:**
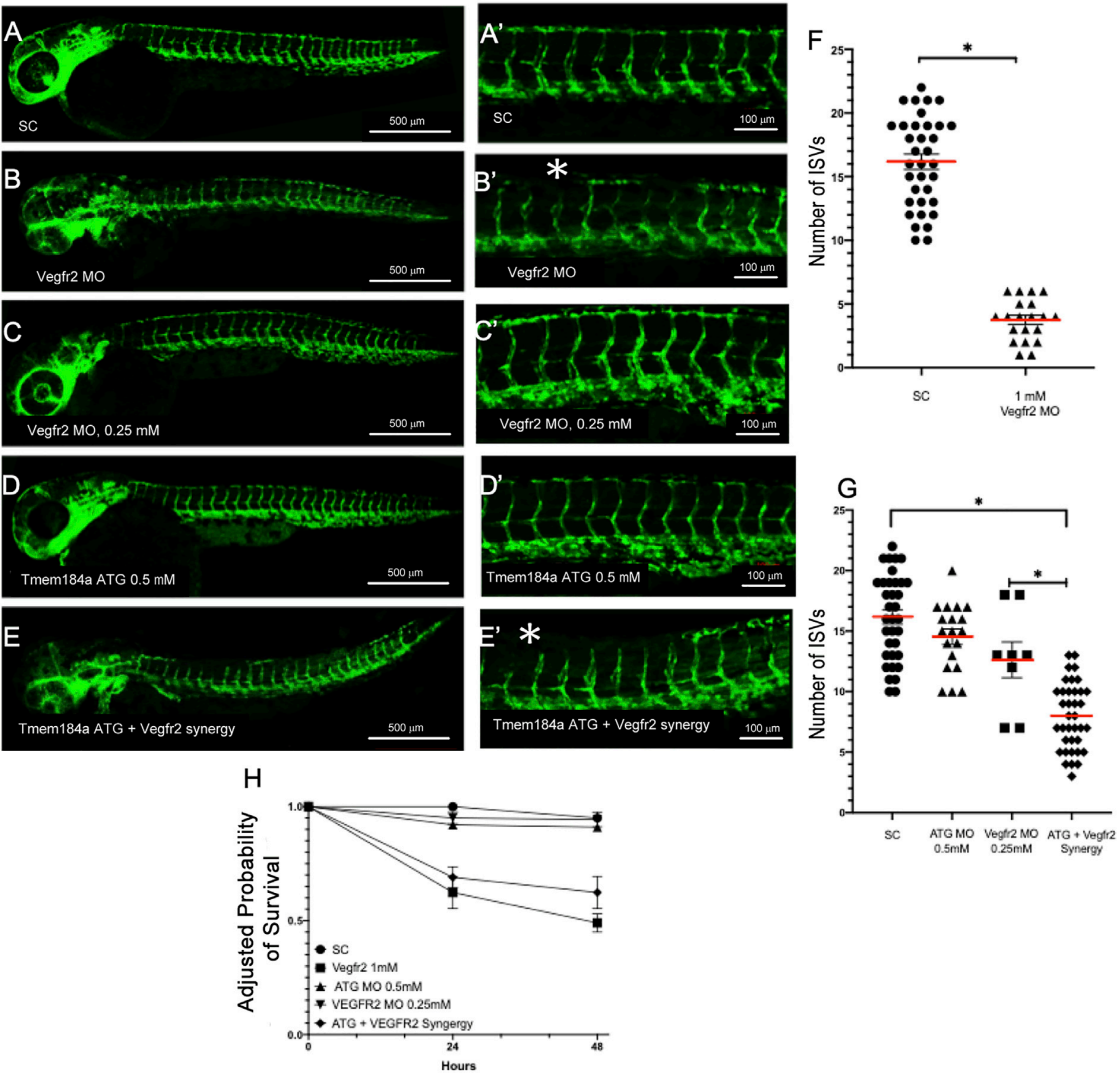
Tmem184a and Vegfr2 Function Synergistically to Modulate Angiogenesis. **(A,A′)** Standard control MO injected embryos (n = 35). **(B,B′)** Vegfr2 MO injected embryos (n = 20). Asterisks indicate ISVs that have failed to complete growth. **(C,C′)** Embryos injected with the subthreshold concentration of the Tmem184a ATG MO (0.50 mM) (n = 14). **(D,D′)** Embryos injected with the subthreshold concentration of the Vegfr2 MO (0.25 mM) (n = 8). **(E,E′)** 48 hpf embryos co-injected with both subthreshold concentrations of the Tmem184a ATG and Vegfr2 MOs (n = 37). Asterisks indicate ISVs that failed to complete growth. **(F)** This graph illustrates the mean number of intact ISVs ± SEM in 1 mm Vegfr2 and SC MOs. *p* < 0.01. **(G)** The graph is the mean number of intact ISVs ± SEM in subthreshold injected Tmem184a ATG and Vegfr2 MOs as compared to both subthreshold MOs. *p* < 0.01. **(H)** Embryo survival for all treatments illustrated relative to 24 h survival of SC embryos.

Angiogenesis is driven by VEGF binding to its receptor (Vegfr2). Vegfr2b mutants have known defects in angiogenesis and exhibit shorter ISVs or vessels that fail to sprout at all. This phenotype is recapitulated by MO-mediated knockdown of Vegfr2 as well (e.g., Figures 5A vs 5B). We hypothesized that Tmem184a interacting with HSPGs might cooperate with Vegfr2 signaling. We utilized a previously characterized Vegfr2b MO to test if Tmem184a and Vegfr2b function in a common pathway to influence angiogenesis (Figures 5A–E). We first demonstrated that we could recapitulate the previously published *vegfr2b* phenotypes. Embryos injected with the Vegfr2 MO had only 4 intact ISVs on average at 48 hpf as compared to the SC MO, which had 16 intact ISVs on average (Figure 5F). We next tested if Vegfr2 and Tmem184a work together in a common molecular pathway by testing for synergistic effects of the two MOs (Figure 5F). The sub-threshold dose of the Vegfr2 MO (0.25 mM) was then co-injected into embryos at the 1 cell stage along with the Tmem184a ATG MO at 0.50 mM. At these concentrations, neither MO alone has much effect on survival or ISV completion at 48 hpf (Figure 5G). The survival of the co-injected embryos was lower than the additive effect of the two subthreshold MOs (Figure 5H). And, we found that the co-injected embryos exhibited significantly fewer intact ISVs as compared to the additive effects of the two MOs (Figure 5G). Together these findings suggest that Tmem184a and Vegfr2 are acting in a common pathway to modulate angiogenesis.

### Tmem184a knockdown causes an increase in endothelial cell proliferation

**FIGURE 6:**
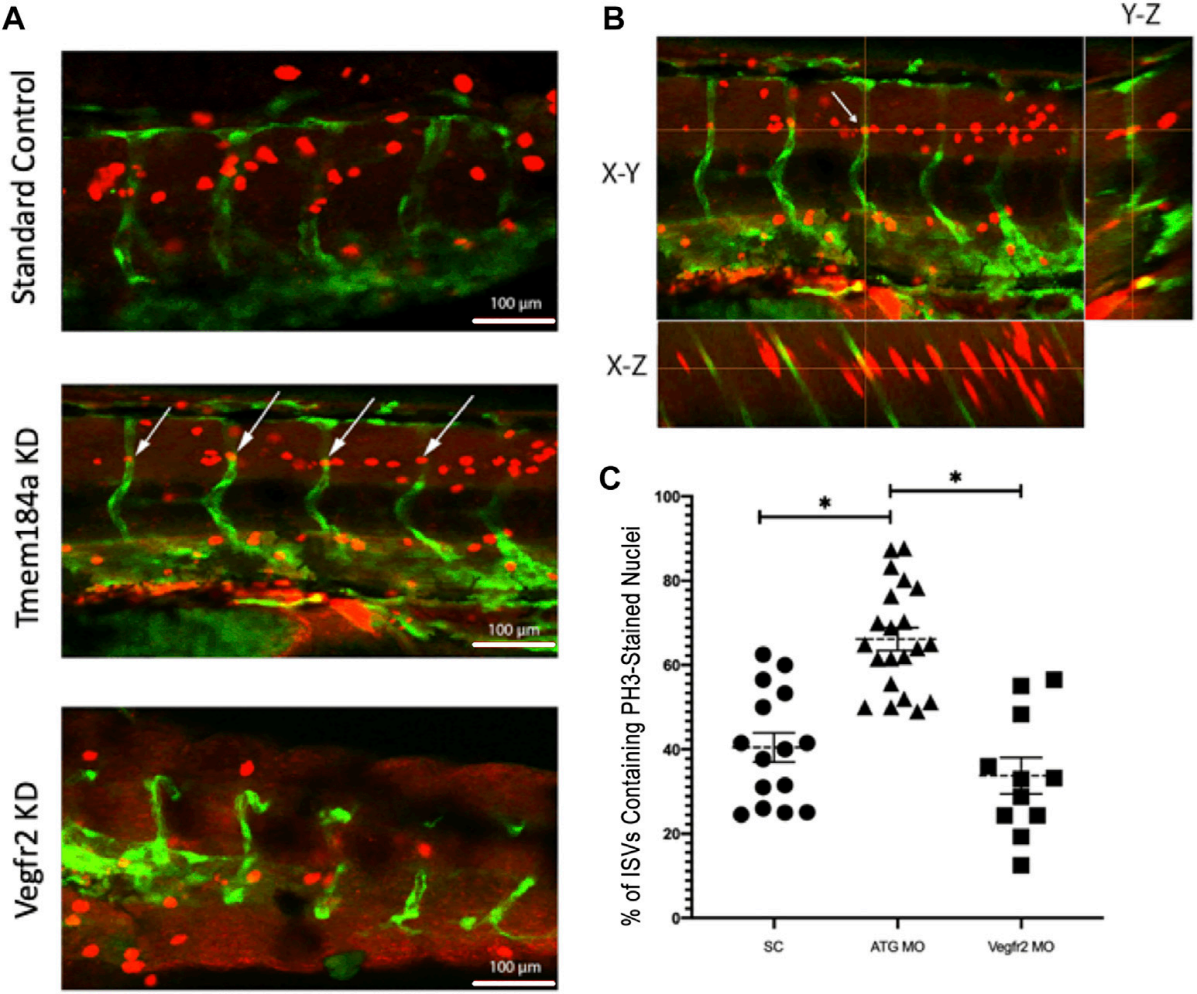
Tmem184a KD Causes an Increase in Cell Proliferation. **(A)** Example images of PH3 staining in SC, Tmem184a, and Vegfr2 MO knockdown embryos at 48 hpf. White arrows indicate where the PH3 staining co-localizes with the GFP in the vasculature of the ATG MO-treated embryo, highlighted in yellow. Z-stack images were rotated to confirm nuclear PH3 in the EC. Embryos injected with the Tmem184a MO (n = 21) exhibit an increase in the percentage of ISVs that contain an PH3 stained nucleus compared to either SC (n = 21) or Vegfr2 (n = 11) injected embryos. **(B)** One PH3 nucleus in an ISV is shown in all planes. Cross-hairs intersect at the stained ISV localized nucleus. **(C)** The graph illustrates the percentage of ISVs per embryo containing an PH3-stained nucleus. *p* < 0.001.

Angiogenesis is dependent upon the Vegfr2 signaling cascade in order for ECs to proliferate and form a vessel. Published data indicate a decrease in EC proliferation in *vegfr2b* mutants. A similar change in the number of available ECs might be responsible for the lack of intact ISVs seen in the Tmem184a KD phenotype. However, our previous data in the zebrafish regenerating fin made it clear that KD of Tmem184a leads to an increase in EC proliferation, indicating that Tmem184a is a negative regulator of EC proliferation in regeneration. In order to determine how Tmem184a influences cell proliferation during development we evaluated the number of histone 3 phosphate (PH3)-positive ECs in 48 hpf zebrafish embryos treated for Tmem184a KD. PH3 staining was quantified using Z-stack confocal images to evaluate where PH3 (red) co-localized inside Fli-positive ECs (green) (Figures 6A,B). Co-localization was evident by the appearance of yellow signal in ECs. We found that Tmem184a KD embryos exhibited an increase in PH3 staining in ISVs as compared to the SC, and therefore an increase in EC proliferation compared to controls (Figure 6C). Because we determined the PH3 labeled nuclei per ISV, the small number of PH3 nuclei in Vegfr2 MO treated embryos matched the lower number of ISVs in these embryos, consistent with published data indicating that lack of EC proliferation correlated with lack of ISV production in Vegfr2 KD embryos. The observed increase in cell proliferation in our Tmem184a KD embryos strongly suggests that the lack of intact ISVs in Tmem184a KD embryos is not secondary to a lack of cell availability, but rather indicates that these cells are unable to organize into proper vessels. This disorganization is also consistent with the disorganized vasculature observed in Tmem184a KD regenerating vasculature published previously.

### Tmem184a knockdown correlates with a decrease in the expression of VE-cadherin

VE-cadherin, a cell-cell adhesion glycoprotein, is important in the adhesion and junctional permeability of endothelial cells in the developing vasculature. Sprouting of new ISVs and extension dorsally continues as VE-cadherin cell-cell contacts re-organize to contribute to the formation of the DLAV.

**FIGURE 7:**
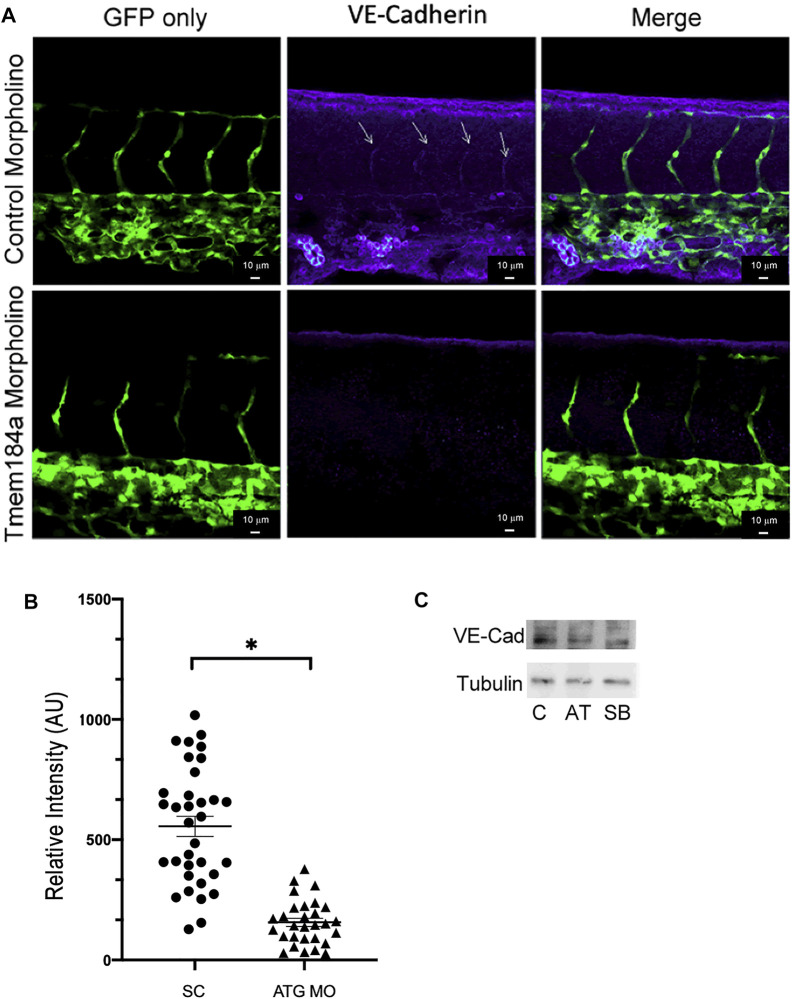
KD of Tmem184a results in decreased levels of VE-cadherin. Embryos were injected with control or ATG-MO for Tmem184a, sorted for GFP positive and fixed at 43 hpf. Embryos were stained with the Anti-zebrafish VE-cadherin antibody as described in Methods and imaged on the Zeiss LSM 880 confocal microscope at 20x. **(A)** Sections in the mid-section of representative embryos are illustrated. Sections illustrated are maximum intensity images as in Methods. Data from 34 Tmem184a KD ISVs and 29 control ISVs from four embryos of each type were analyzed for VE-cadherin intensity across all ISVs in the mid-sections imaged and a graph illustrating relative VE-cadherin is shown in **(B)**. A student’s T test was used to determine significance, *p* < 0.001 **(C)** Western blots of identically treated embryo lysates are shown.

Given that we did not find a lack of EC proliferation to be the cause of our incomplete ISVs, we considered that vessels in our morphant fish may be unable to complete outgrowth because of altered VE-cadherin-modulated outgrowth. We hypothesized that decreased Tmem184a levels might result in decreased VE-cadherin, and that the stalk cells in the sprouting ISV would then be less effective at migration and less able to organize dorsally. In order to test this, we performed immunostaining for VE-cadherin in 43 hpf zebrafish embryos treated for Tmem184a ATG KD and in standard controls. The time was chosen to optimize the visual VE-cadherin difference in ISVs between control and KD embryos. These embryos were analyzed using confocal microscopy with Z-stack imaging to evaluate co-localization of VE-cadherin with the GFP in the vasculature (Figure 7A). Intensity of the VE-cadherin staining was then measured per ISV (Figure 7). We found a decrease in VE-cadherin intensity in the ISVs of the Tmem184a ATG KD embryos as compared to controls (Figure 7B). We confirmed that there was a decrease in VE-cadherin levels in the ATG KD embryos through western blotting of embryo lysates (Figure 7C). Therefore, it is possible that despite the excess EC proliferation, ECs in the Tmem184a KD embryos are less able to organize to form a vessel because of a lack of VE-cadherin adhesions.

## Discussion

**FIGURE 8:**
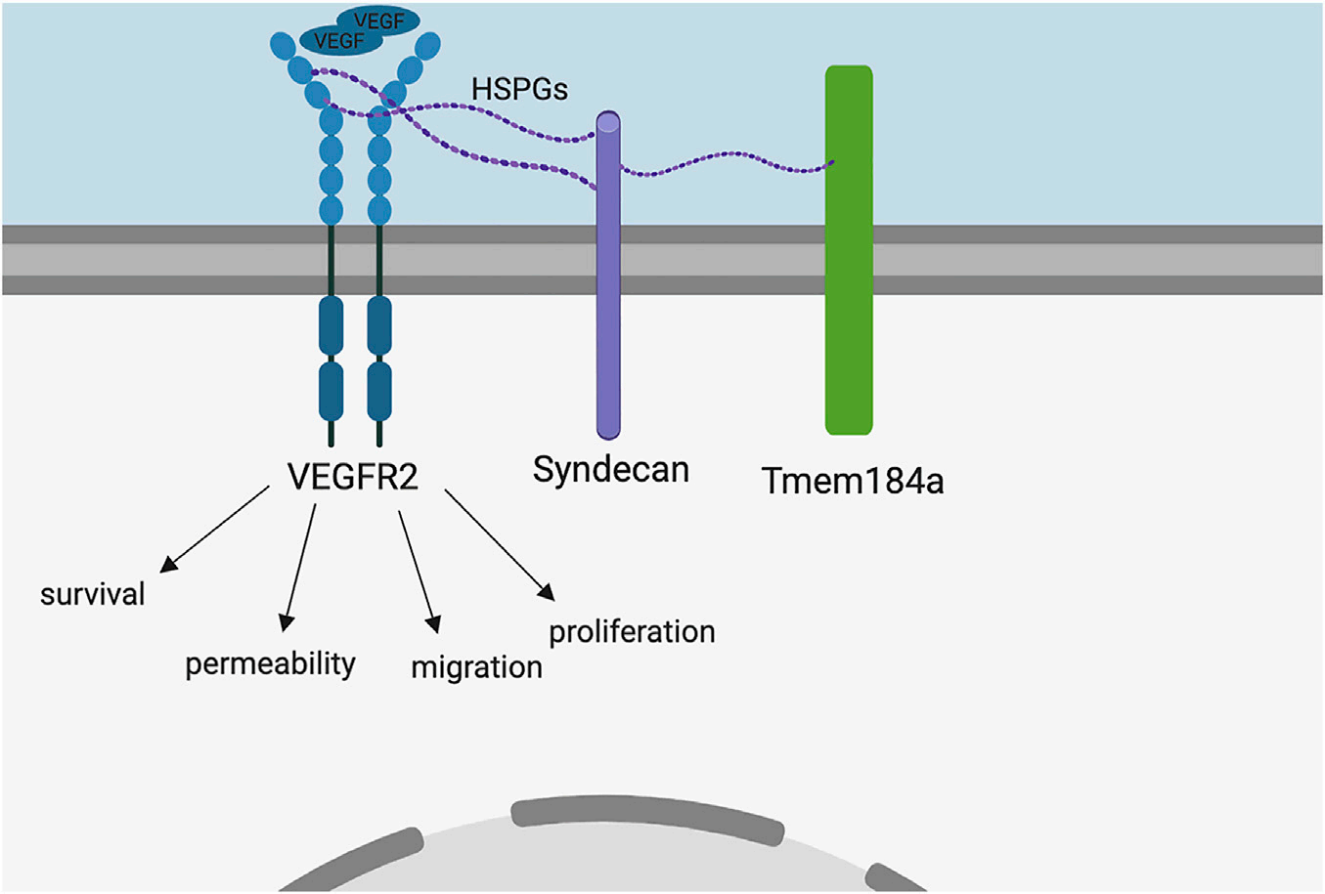
Model. Based on the literature, we hypothesized HS interaction with Tmem184a is involved in modulation of Vegfr2 signal transduction. VEGF binding to Vegfr2 induces a signaling cascade responsible for the EC survival, permeability, migration, and proliferation necessary for angiogenesis. The present study provides evidence supporting this hypothesis.

The heparin receptor, Tmem184a, was identified in vascular ECs (Farwell et al., 2016). Tmem184a is responsible for the anti-proliferative signaling cascades induced by heparin *in vitro* (Gilotti et al., 2014; Pugh et al., 2016; Li et al., 2020). Previous *in vivo* studies show that Tmem184a is required for proper vascular regeneration length in the adult regenerating zebrafish caudal fin (Farwell et al., 2017). Here, we provide evidence that Tmem184a is required for proper vessel growth in the developing embryo as well, and KD of Tmem184a also decreases embryo survival. There are no previous reports of Tmem184a in vascular development for any species. Our data are consistent with the model shown in Figure 8 which identifies a role for this heparin receptor in developmental as well as regenerative angiogenesis. We demonstrate that knocking down Tmem184a expression negatively impacts angiogenesis and supported this observation with a rescue experiment which rescues survival along with ISV development. Our data indicate that in the Tmem184a KD embryos, ISVs begin to sprout at the same time identified in control embryos (e.g., Gore et al., 2012), but complete outgrowth fails, and many ISVs are not complete at 48 hpf. This developmental phenotype resembles our previous findings of regenerative angiogenesis where endothelial cells proliferate, but do not grow out properly. In addition, we see decreased survival, something not possible to observe in the regeneration studies.

Our earlier identification of Tmem184a as a heparin receptor (Farwell et al., 2016; Pugh et al., 2016) suggests that Tmem184a functions through interactions with HS chains. Therefore, we investigated whether Tmem184a was modulating angiogenesis via HSPG chains by synthesizing mRNA that does not contain coding for the putative HS binding domain from the Tmem184a mRNA and injecting the resulting shortened message into the single cell stage embryos. By including a shortened mRNA, the coding in the message results in a truncated protein that does not include a HS/heparin binding site. Our results indicate that the *tmem184a-ΔCT* mRNA has a negative effect on the ISV development, even in embryos where normal Tmem184a is present. This domain is therefore important for modulating angiogenesis. We observed that by increasing the dose of the *tmem184a-ΔCT* mRNA, there was a greater effect on the ISV development and survival levels were lower. The effect is not seen in the full-length Tmem184a mRNA. Double injections with the *tmem184a-ΔCT* mRNA and Tmem184a SB MO, also supported the need for the HS chain interactions in Tmem184a modulation of angiogenesis. The increase in severity of the SB MO phenotype by *tmem184a-ΔCT* mRNA implies that Tmem184a is involved in complex formation, presumably at least partly through the putative HS binding domain and HSPG interactions. This additional phenotype identified in the *tmem184a-ΔCT* mRNA treated embryos implies a more complicated function than one where Tmem184a simply binds to HS chains. At the minimum, the remainder of the Tmem184a protein must form dimers as suggested by heparin binding assays (Pugh et al., 2016) or interact with other proteins such that the presence of Tmem184a without the putative heparin binding domain competes with the wild type Tmem184a in the embryos without MO KD causing both the same outcomes as the KD embryos and the increase in spacing between ISVs. This phenotype is not just more severe than the SB MO KD treatment, because the ATG MO and the SB MO alone both result in similar loss of complete ISVs and a decrease in survival but do not increase in distance between the ISVs. Possibly, the signals that lead to sprouting accumulate more slowly in the *tmem184a-ΔCT* mRNA-treated embryos, resulting in delays in the ISV sprouting.

The incomplete ISV phenotype observed with Tmem184a KD is similar to what has been observed in some Vegfr2 mutations (Gavard and Gutkind, 2006; Covassin et al., 2009) where only partial ISV outgrowth occurs. In complete VEGF/Vegfr2 deletion, some early vascular development still occurs. However, these VEGF deletion embryos have more severe ISV effects than seen in Tmem184a KD embryos, but similar to those in our Vegfr2 KD embryos (Habeck et al., 2002). We tested our hypothesis that Tmem184a modulates angiogenesis through the Vegfr2 pathway through a synergy experiment. We find that Tmem184a synergizes with Vegfr2 signaling, identifying a potential mechanism by which Tmem184a modulates angiogenesis. Because Vegfr2 signaling promotes the EC proliferation, migration, tissue permeability, and survival that are all required for angiogenesis and complete circulatory development (Koch et al., 2011), we suggest that Tmem184a is acting in one or more of these Vegfr2-induced outcomes to modulate proper vessel development. It is intriguing to consider the fact that co-receptors (Lanahan et al., 2013), different VEGF ligands (Fearnley et al., 2016), and other mechanisms that modulate the internalization process (Lanahan et al., 2010; Lanahan et al., 2014), also impact Vegfr2 signaling outcomes. For example, Plcg1 (downstream from Vegfr2) defects are as severe as the most severe VEGF mutants (Covassin et al., 2009). Therefore, KD of Tmem184a could impede normal development by limiting one or more of the outcomes and/or by altering the balance between the various outcomes.

We examined whether Tmem184a KD had an impact on EC proliferation, leaving fewer cells to form a complete vessel, or resulting in too many cells to organize properly, as we observed in Tmem184a KD in fin regeneration (Farwell et al., 2017). Our findings show that Tmem184a KD increases EC proliferation as it does in cell culture and zebrafish fin regenerating models. This suggests that cell availability is not an issue for vessel development after Tmem184a KD, but that the available cells are unable to organize into proper vessels during angiogenesis. Therefore, Tmem184a does not completely match Vegfr2 signaling, but rather Tmem184a either modulates one or more outcomes of Vegfr2 signaling or is required for a sub-set of the outcomes, acting to achieve balance between the outcomes.

We therefore examined whether Tmem184a KD altered VE-cadherin, a cell adhesion protein, the internalization of which is downstream of Vegfr2 and critical for angiogenesis. We find that when Tmem184a is knocked down, VE-cadherin, is also decreased in ISVs. Without proper adhesions, it is possible that ECs may be less able to migrate dorsally and correctly stack on one another to form a complete ISV. VE-cadherin is critical for organization of cell-cell interactions required to reorganize the actin fibers in the developing stalk, though longitudinal vessels formed by vasculogenesis appear to develop normally in the absence of VE-cadherin (Sauteur et al., 2014). VE-cadherin is involved in initial contacts that mediate the cell-cell junctions in anastomosis leading to the formation of the DLAV (Sauteur et al., 2017). It is unclear whether the VE-cadherin decrease is the only outcome of Tmem184a KD. The mechanism by which the KD of Tmem184a results in lowered levels of VE-cadherin remains to be determined. VE-cadherin phosphorylation and dephosphorylation are critical for modulation of adherens junction maturation and permeability (Carra et al., 2012; Sidibe et al., 2014; Sidibe and Imhof, 2014). Thus, signaling through Tmem184a could impact VE-cadherin phosphorylation leading to the decreased levels in response to VE-cadherin endocytosis. Earlier studies (Best and Adams, 2009) suggest a potential role for Tmem184a in vesicle trafficking, one mechanism by which the loss of Tmem184a could impact VE-cadherin levels. It will be critical to determine whether the decrease in VE-cadherin levels is the mechanism responsible for the lower survival and lack of ISV completion in Tmem184a KD embryos or is simply evidence of the problem and not part of a mechanism.

We infer from these results that Tmem184a interacts with the Vegf receptor through HS chains provided by an HSPG given that HSPGs typically have three HS chains per HSPG, and these chains spread out in different directions making it probable that they can interact with different proteins. It is possible that a different relationship between the Vegf receptor and Tmem184a might exist instead. Further investigation involving which HSPG is connecting Tmem184a to Vegf and Vegfr2 will be crucial to determine the necessary HSPG(s). Syndecan (Sdc) -2, -3, and -4 are HSPGs expressed in zebrafish and have been illustrated to play roles in cellular signaling and the activation of downstream pathways. Sdc2, for instance, participates in blood vessel patterning downstream of axial vessel differentiation (Chen et al., 2004) and is necessary for vascular development (Chen et al., 2004; Corti et al., 2019). The absence of Sdc2 results in failed or abnormally developed ISVs. The HS chains attached to Sdc2 protein can bind to VEGF and facilitate the interaction with its receptor Vegfr2, in turn helping to activate angiogenesis (Couchman et al., 2016). Many others have found evidence for Sdc4 involvement in angiogenesis (Echtermeyer et al., 2001). In particular, migration, flow responses and lymphatic angiogenesis seem to depend on Sdc4 (Baeyens et al., 2014; Wang et al., 2016) and Sdc4 plays a role in modulating migration in FGF angiogenic signaling (Elfenbein et al., 2009). Intriguingly, Sdc4 has recently been identified as critical for modulating VE-cadherin internalization and recycling during angiogenesis (De Rossi et al., 2021). It is also possible that multiple HSPGs interact with Tmem184a and are coordinately involved in modulating angiogenesis. Additional studies will be necessary to resolve these possibilities.

In conclusion, we have described how KD of Tmem184a interferes with proper vessel development in an *in vivo* model through collaborations with the VEGF receptor, Vegfr2, by increasing cell proliferation and decreasing cell adhesion proteins. The process appears to require interaction between Tmem184a and HSPGs. Further understanding of the mechanisms by which Tmem184a modulates angiogenesis may have implications in developing therapies for human angiogenic diseases.

## Data Availability

The original contributions presented in the study are included in the article/Supplementary Material, further inquiries can be directed to the corresponding author.

## References

[B1] Ando K. Fukuhara S. Izumi N. Nakajima H. Fukui H. Kelsh R. N. (2016). Clarification of mural cell coverage of vascular endothelial cells by live imaging of zebrafish. Development 143 (8), 1328–1339. 10.1242/dev.132654 26952986 PMC4852519

[B2] Baeyens N. Mulligan-Kehoe M. J. Corti F. Simon D. D. Ross T. D. Rhodes J. M. (2014). Syndecan 4 is required for endothelial alignment in flow and atheroprotective signaling. Proc. Natl. Acad. Sci. U. S. A. 111 (48), 17308–17313. 10.1073/pnas.1413725111 25404299 PMC4260558

[B3] Bahary N. Goishi K. Stuckenholz C. Weber G. Leblanc J. Schafer C. A. (2007). Duplicate VegfA genes and orthologues of the KDR receptor tyrosine kinase family mediate vascular development in the zebrafish. Blood 110 (10), 3627–3636. 10.1182/blood-2006-04-016378 17698971 PMC2077312

[B4] Banerji R. Eble D. M. Iovine M. K. Skibbens R. V. (2016). Esco2 regulates cx43 expression during skeletal regeneration in the zebrafish fin. Dev. Dyn. 245 (1), 7–21. 10.1002/dvdy.24354 26434741

[B5] Beauvais D. M. Ell B. J. McWhorter A. R. Rapraeger A. C. (2009). Syndecan-1 regulates alphavbeta3 and alphavbeta5 integrin activation during angiogenesis and is blocked by synstatin, a novel peptide inhibitor. J. Exp. Med. 206 (3), 691–705. 10.1084/jem.20081278 19255147 PMC2699122

[B6] Bernfield M. Gotte M. Park P. W. Reizes O. Fitzgerald M. L. Lincecum J. (1999). Functions of cell surface heparan sulfate proteoglycans. Annu. Rev. Biochem. 68, 729–777. 10.1146/annurev.biochem.68.1.729 10872465

[B7] Best D. Adams I. R. (2009). Sdmg1 is a component of secretory granules in mouse secretory exocrine tissues. Dev. Dyn. 238 (1), 223–231. 10.1002/dvdy.21827 19097053

[B8] Blum Y. Belting H. G. Ellertsdottir E. Herwig L. Luders F. Affolter M. (2008). Complex cell rearrangements during intersegmental vessel sprouting and vessel fusion in the zebrafish embryo. Dev. Biol. 316 (2), 312–322. 10.1016/j.ydbio.2008.01.038 18342303

[B9] Carra S. Foglia E. Cermenati S. Bresciani E. Giampietro C. Lora Lamia C. (2012). Ve-ptp modulates vascular integrity by promoting adherens junction maturation. PLoS One 7 (12), e51245. 10.1371/journal.pone.0051245 23251467 PMC3522677

[B10] Chen E. Hermanson S. Ekker S. C. (2004). Syndecan-2 is essential for angiogenic sprouting during zebrafish development. Blood 103 (5), 1710–1719. 10.1182/blood-2003-06-1783 14592839

[B11] Childs S. Chen J. N. Garrity D. M. Fishman M. C. (2002). Patterning of angiogenesis in the zebrafish embryo. Development 129 (4), 973–982. 10.1242/dev.129.4.973 11861480

[B12] Chiodelli P. Bugatti A. Urbinati C. Rusnati M. (2015). Heparin/heparan sulfate proteoglycans glycomic interactome in angiogenesis: Biological implications and therapeutical use. Molecules 20 (4), 6342–6388. 10.3390/molecules20046342 25867824 PMC6272510

[B13] Corti F. Wang Y. Rhodes J. M. Atri D. Archer-Hartmann S. Zhang J. (2019). N-terminal syndecan-2 domain selectively enhances 6-O heparan sulfate chains sulfation and promotes VEGFA165-dependent neovascularization. Nat. Commun. 10 (1), 1562. 10.1038/s41467-019-09605-z 30952866 PMC6450910

[B14] Couchman J. R. Multhaupt H. Sanderson R. D. (2016). Recent insights into cell surface heparan sulphate proteoglycans and cancer. F1000Res 5, F1000. 10.12688/f1000research.8543.1 PMC4930033 27408707

[B15] Covassin L. D. Siekmann A. F. Kacergis M. C. Laver E. Moore J. C. Villefranc J. A. (2009). A genetic screen for vascular mutants in zebrafish reveals dynamic roles for Vegf/Plcg1 signaling during artery development. Dev. Biol. 329 (2), 212–226. 10.1016/j.ydbio.2009.02.031 19269286 PMC2791107

[B16] De Rossi G. Vahatupa M. Cristante E. Arokiasamy S. Liyanage S. E. May U. (2021). Pathological angiogenesis requires syndecan-4 for efficient VEGFA-induced VE-cadherin internalization. Arterioscler. Thromb. Vasc. Biol. 41 (4), 1374–1389. 10.1161/ATVBAHA.121.315941 33596666 PMC7613699

[B17] Echtermeyer F. Streit M. Wilcox-Adelman S. Saoncella S. Denhez F. Detmar M. (2001). Delayed wound repair and impaired angiogenesis in mice lacking syndecan-4. J. Clin. Invest. 107 (2), R9–R14. 10.1172/JCI10559 11160142 PMC199172

[B18] Elfenbein A. Rhodes J. M. Meller J. Schwartz M. A. Matsuda M. Simons M. (2009). Suppression of RhoG activity is mediated by a syndecan 4-synectin-RhoGDI1 complex and is reversed by PKCalpha in a Rac1 activation pathway. J. Cell Biol. 186 (1), 75–83. 10.1083/jcb.200810179 19581409 PMC2712988

[B19] Farwell S. L. Kanyi D. Hamel M. Slee J. B. Miller E. A. Cipolle M. D. (2016). Heparin decreases in tumor necrosis factor α (TNFα)-induced endothelial stress responses require transmembrane protein 184A and induction of dual specificity phosphatase 1. J. Biol. Chem. 291 (10), 5342–5354. 10.1074/jbc.M115.681288 26769965 PMC4777865

[B20] Farwell S. L. N. Reylander K. G. Iovine M. K. Lowe-Krentz L. J. (2017). Novel heparin receptor transmembrane protein 184a regulates angiogenesis in the adult zebrafish caudal fin. Front. Physiol. 8, 671. 10.3389/fphys.2017.00671 28936181 PMC5594097

[B21] Fearnley G. W. Smith G. A. Abdul-Zani I. Yuldasheva N. Mughal N. A. Homer-Vanniasinkam S. (2016). VEGF-A isoforms program differential VEGFR2 signal transduction, trafficking and proteolysis. Biol. Open 5 (5), 571–583. 10.1242/bio.017434 27044325 PMC4874356

[B22] Gavard J. Gutkind J. S. (2006). VEGF controls endothelial-cell permeability by promoting the beta-arrestin-dependent endocytosis of VE-cadherin. Nat. Cell Biol. 8 (11), 1223–1234. 10.1038/ncb1486 17060906

[B23] Geudens I. Gerhardt H. (2011). Coordinating cell behaviour during blood vessel formation. Development 138 (21), 4569–4583. 10.1242/dev.062323 21965610

[B24] Gilotti A. C. Nimlamool W. Pugh R. Slee J. B. Barthol T. C. Miller E. A. (2014). Heparin responses in vascular smooth muscle cells involve cGMP-dependent protein kinase (PKG). J. Cell. Physiol. 229 (12), 2142–2152. 10.1002/jcp.24677 24911927 PMC4149598

[B25] Gore A. V. Monzo K. Cha Y. R. Pan W. Weinstein B. M. (2012). Vascular development in the zebrafish. Cold Spring Harb. Perspect. Med. 2 (5), a006684. 10.1101/cshperspect.a006684 22553495 PMC3331685

[B26] Habeck H. Odenthal J. Walderich B. Maischein H. Schulte-Merker S. (2002). Analysis of a zebrafish VEGF receptor mutant reveals specific disruption of angiogenesis. Curr. Biol. 12 (16), 1405–1412. 10.1016/s0960-9822(02)01044-8 12194822

[B27] Hasan S. S. Tsaryk R. Lange M. Wisniewski L. Moore J. C. Lawson N. D. (2017). Endothelial Notch signalling limits angiogenesis via control of artery formation. Nat. Cell Biol. 19 (8), 928–940. 10.1038/ncb3574 28714969 PMC5534340

[B28] Helker C. S. Schuermann A. Karpanen T. Zeuschner D. Belting H. G. Affolter M. (2013). The zebrafish common cardinal veins develop by a novel mechanism: Lumen ensheathment. Development 140 (13), 2776–2786. 10.1242/dev.091876 23698350

[B29] Herbert S. P. Stainier D. Y. (2011). Molecular control of endothelial cell behaviour during blood vessel morphogenesis. Nat. Rev. Mol. Cell Biol. 12 (9), 551–564. 10.1038/nrm3176 21860391 PMC3319719

[B30] Higashijima S. (2008). Transgenic zebrafish expressing fluorescent proteins in central nervous system neurons. Dev. Growth Differ. 50 (6), 407–413. 10.1111/j.1440-169X.2008.01023.x 18445066

[B31] Johns S. C. Yin X. Jeltsch M. Bishop J. R. Schuksz M. El Ghazal R. (2016). Functional importance of a proteoglycan coreceptor in pathologic lymphangiogenesis. Circ. Res. 119 (2), 210–221. 10.1161/CIRCRESAHA.116.308504 27225479 PMC4938725

[B32] Koch S. Tugues S. Li X. Gualandi L. Claesson-Welsh L. (2011). Signal transduction by vascular endothelial growth factor receptors. Biochem. J. 437 (2), 169–183. 10.1042/BJ20110301 21711246

[B33] Lanahan A. A. Hermans K. Claes F. Kerley-Hamilton J. S. Zhuang Z. W. Giordano F. J. (2010). VEGF receptor 2 endocytic trafficking regulates arterial morphogenesis. Dev. Cell 18 (5), 713–724. 10.1016/j.devcel.2010.02.016 20434959 PMC2875289

[B34] Lanahan A. A. Lech D. Dubrac A. Zhang J. Zhuang Z. W. Eichmann A. (2014). PTP1b is a physiologic regulator of vascular endothelial growth factor signaling in endothelial cells. Circulation 130 (11), 902–909. 10.1161/CIRCULATIONAHA.114.009683 24982127 PMC6060619

[B35] Lanahan A. Zhang X. Fantin A. Zhuang Z. Rivera-Molina F. Speichinger K. (2013). The neuropilin 1 cytoplasmic domain is required for VEGF-A-dependent arteriogenesis. Dev. Cell 25 (2), 156–168. 10.1016/j.devcel.2013.03.019 23639442 PMC3774154

[B36] Lawson N. D. Weinstein B. M. (2002). *In vivo* imaging of embryonic vascular development using transgenic zebrafish. Dev. Biol. 248 (2), 307–318. 10.1006/dbio.2002.0711 12167406

[B37] Leslie J. D. Ariza-McNaughton L. Bermange A. L. McAdow R. Johnson S. L. Lewis J. (2007). Endothelial signalling by the Notch ligand Delta-like 4 restricts angiogenesis. Development 134 (5), 839–844. 10.1242/dev.003244 17251261

[B38] Li Y. Talotta-Altenburg L. M. Silimperi K. A. Ciabattoni G. O. Lowe-Krentz L. J. (2020). Endothelial nitric oxide synthase activation is required for heparin receptor effects on vascular smooth muscle cells. Am. J. Physiol. Cell Physiol. 318 (3), C463–C475. 10.1152/ajpcell.00284.2018 31891520 PMC7099515

[B39] Liang D. Xu X. Chin A. J. Balasubramaniyan N. V. Teo M. A. Lam T. J. (1998). Cloning and characterization of vascular endothelial growth factor (VEGF) from zebrafish, *Danio rerio* . Biochim. Biophys. Acta 1397 (1), 14–20. 10.1016/s0167-4781(97)00233-9 9545518

[B40] Nunes S. S. Outeiro-Bernstein M. A. Juliano L. Vardiero F. Nader H. B. Woods A. (2008). Syndecan-4 contributes to endothelial tubulogenesis through interactions with two motifs inside the pro-angiogenic N-terminal domain of thrombospondin-1. J. Cell. Physiol. 214 (3), 828–837. 10.1002/jcp.21281 17879962

[B41] Pugh R. J. Slee J. B. Farwell S. L. Li Y. Barthol T. Patton W. A. (2016). Transmembrane protein 184A is a receptor required for vascular smooth muscle cell responses to heparin. J. Biol. Chem. 291 (10), 5326–5341. 10.1074/jbc.M115.681122 26769966 PMC4777864

[B42] Rawls J. F. Frieda M. R. McAdow A. R. Gross J. P. Clayton C. M. Heyen C. K. (2003). Coupled mutagenesis screens and genetic mapping in zebrafish. Genetics 163 (3), 997–1009. 10.1093/genetics/163.3.997 12663538 PMC1462478

[B43] Santoro M. M. Pesce G. Stainier D. Y. (2009). Characterization of vascular mural cells during zebrafish development. Mech. Dev. 126 (8-9), 638–649. 10.1016/j.mod.2009.06.1080 19539756 PMC2732398

[B44] Sarrazin S. Lamanna W. C. Esko J. D. (2011). Heparan sulfate proteoglycans. Cold Spring Harb. Perspect. Biol. 3 (7), a004952. 10.1101/cshperspect.a004952 21690215 PMC3119907

[B45] Sauteur L. Affolter M. Belting H. G. (2017). Distinct and redundant functions of Esama and VE-cadherin during vascular morphogenesis. Development 144 (8), 1554–1565. 10.1242/dev.140038 28264837

[B46] Sauteur L. Krudewig A. Herwig L. Ehrenfeuchter N. Lenard A. Affolter M. (2014). Cdh5/VE-cadherin promotes endothelial cell interface elongation via cortical actin polymerization during angiogenic sprouting. Cell Rep. 9 (2), 504–513. 10.1016/j.celrep.2014.09.024 25373898

[B47] Schnabel D. Castillo-Robles J. Lomeli H. (2019). Protein purification and western blot detection from single zebrafish embryo. Zebrafish 16 (6), 505–507. 10.1089/zeb.2019.1761 31408407

[B48] Schultz V. Suflita M. Liu X. Zhang X. Yu Y. Li L. (2017). Heparan sulfate domains required for fibroblast growth factor 1 and 2 signaling through fibroblast growth factor receptor 1c. J. Biol. Chem. 292 (6), 2495–2509. 10.1074/jbc.M116.761585 28031461 PMC5313116

[B49] Sidibe A. Imhof B. A. (2014). VE-Cadherin phosphorylation decides: Vascular permeability or diapedesis. Nat. Immunol. 15 (3), 215–217. 10.1038/ni.2825 24549064

[B50] Sidibe A. Polena H. Pernet-Gallay K. Razanajatovo J. Mannic T. Chaumontel N. (2014). VE-cadherin Y685F knock-in mouse is sensitive to vascular permeability in recurrent angiogenic organs. Am. J. Physiol. Heart Circ. Physiol. 307 (3), H455–H463. 10.1152/ajpheart.00774.2013 24858856

[B51] Siekmann A. F. Lawson N. D. (2007). Notch signalling limits angiogenic cell behaviour in developing zebrafish arteries. Nature 445 (7129), 781–784. 10.1038/nature05577 17259972

[B52] Simons M. Gordon E. Claesson-Welsh L. (2016). Mechanisms and regulation of endothelial VEGF receptor signalling. Nat. Rev. Mol. Cell Biol. 17 (10), 611–625. 10.1038/nrm.2016.87 27461391

[B53] Suchting S. Freitas C. le Noble F. Benedito R. Breant C. Duarte A. (2007). The Notch ligand Delta-like 4 negatively regulates endothelial tip cell formation and vessel branching. Proc. Natl. Acad. Sci. U. S. A. 104 (9), 3225–3230. 10.1073/pnas.0611177104 17296941 PMC1805603

[B54] Villefranc J. A. Nicoli S. Bentley K. Jeltsch M. Zarkada G. Moore J. C. (2013). A truncation allele in vascular endothelial growth factor c reveals distinct modes of signaling during lymphatic and vascular development. Development 140 (7), 1497–1506. 10.1242/dev.084152 23462469 PMC3596992

[B55] Wang Y. Baeyens N. Corti F. Tanaka K. Fang J. S. Zhang J. (2016). Syndecan 4 controls lymphatic vasculature remodeling during mouse embryonic development. Development 143 (23), 4441–4451. 10.1242/dev.140129 27789626 PMC5201046

[B56] Whitesell T. R. Kennedy R. M. Carter A. D. Rollins E. L. Georgijevic S. Santoro M. M. (2014). An α-smooth muscle actin (acta2/αsma) zebrafish transgenic line marking vascular mural cells and visceral smooth muscle cells. PLoS One 9 (3), e90590. 10.1371/journal.pone.0090590 24594685 PMC3940907

[B57] Wiens K. M. Lee H. L. Shimada H. Metcalf A. E. Chao M. Y. Lien C. L. (2010). Platelet-derived growth factor receptor beta is critical for zebrafish intersegmental vessel formation. PLoS One 5 (6), e11324. 10.1371/journal.pone.0011324 20593033 PMC2892519

