# Supplementary material for: Involvement of transmembrane protein 184a during angiogenesis in zebrafish embryos
Source: Front Physiol. 2022 Sep 2;13:845407. doi: 10.3389/fphys.2022.845407 (PMC9478037; doi:10.3389/fphys.2022.845407)

Supplementary figure 1. **Confirmation of Tmem184a KD.** Two MOs for Tmem184a sufficiently KD expression of the Tmem184a protein. (A) Embryos injected with the subthreshold concentration of the Tmem184a ATG MO (0.75mM) (n=14). (B) Embryos injected with the subthreshold concentration of the Tmem184a SB MO (0.75mM) (n=11). (C) 48 hpf embryos co-injected with both subthreshold concentrations of the Tmem184a ATG and Tmem184a SB MOs (n=10). Asterisks indicate ISVs that failed to complete outgrowth. (D). KD with SB and ATG together is depicted in the graph (E) as the mean number of intact ISVs +/- SEM P<0.001. (F) Embryo survival for all treatments shown as compared to SC embryo survival at 24 hrs.

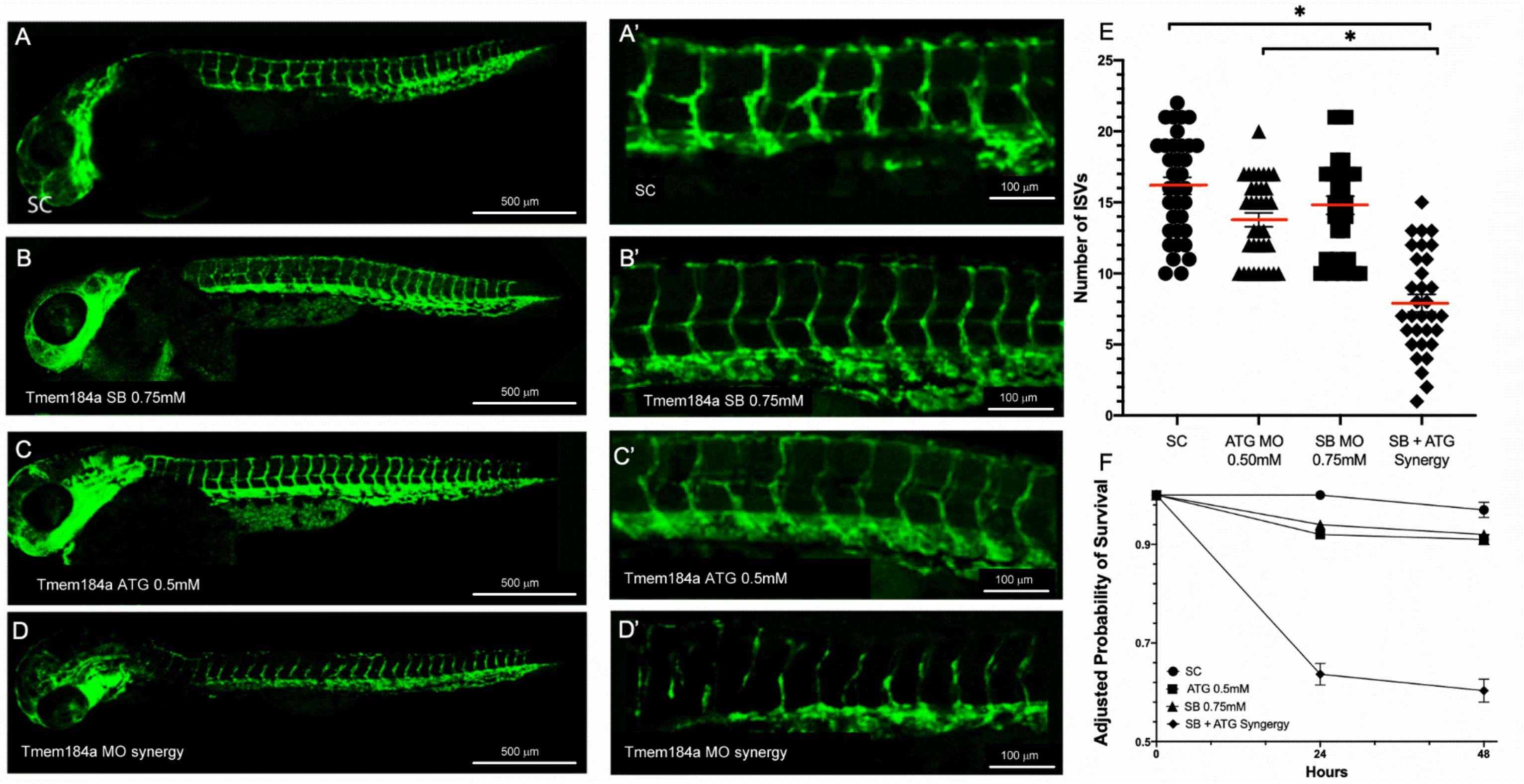

Supplement: Supplementary file 1 [file DataSheet1.PDF]
